# Size Effect of Ordered Mesoporous Carbon Nanospheres for Anodes in Li-Ion Battery

**DOI:** 10.3390/nano5042348

**Published:** 2015-12-18

**Authors:** Pei-Yi Chang, Kartick Bindumadhavan, Ruey-An Doong

**Affiliations:** 1 Department of Biomedical Engineering and Environmental Sciences, National Tsing Hua University, Hsinchu 30013, Taiwan; E-Mail: yirande@gmail.com; 2 Institute of Environmental Engineering, National Chiao Tung University, Hsinchu 30010, Taiwan; E-Mail: radoong@nctu.edu.tw

**Keywords:** ordered mesoporous carbon nanospheres (OMCS), particle size distribution, Li ion batteries (LIBs), rate capability

## Abstract

The present work demonstrates the application of various sizes of ordered mesoporous carbon nanospheres (OMCS) with diameters of 46–130 nm as an active anode material for Li-ion batteries (LIB). The physical and chemical properties of OMCS have been evaluated by performing scanning electron microscopy (SEM), transmission electron microscopy (TEM), N_2_ adsorption-desorption analysis; small-angle scattering system (SAXS) and X-ray diffraction (XRD). The electrochemical analysis of using various sizes of OMCS as anode materials showed high capacity and rate capability with the specific capacity up to 560 mA·h·g^−1^ at 0.1 *C* after 85 cycles. In terms of performance at high current rate compared to other amorphous carbonaceous materials; a stable and extremely high specific capacity of 240 mA·h·g^−1^ at 5 *C* after 15 cycles was achieved. Such excellent performance is mainly attributed to the suitable particle size distribution of OMCS and intimate contact between OMCS and conductive additives; which can be supported from the TEM images. Results obtained from this study clearly indicate the excellence of size distribution of highly integrated mesoporous structure of carbon nanospheres for LIB application.

## 1. Introduction

Recent decades have witnessed unprecedented interest toward the development of alternate energy devices due to continuous depletion of conventional energy sources. However, the development of stable and portable energy generation and storage systems always remains a challenging task [1,2]. Among the alternate energy devices, rechargeable Li-ion batteries (LIBs) have been considered as one of the trusted systems [3,4,5]. They have been invariably applied in electronic devices, such as hybrid electric vehicles, smartphone, and notebook and tablet personal computers [6,7,8,9,10]. The performance of these LIBs is largely dependent on the characteristics of anode, cathode and electrolyte [11]. Additionally, carbonaceous intercalation compounds are one of the first reported active materials for LIBs owing to their abundant source and relevant electronic (in-plane) conductivity for charge storage [12,13,14,15]. Recently, a great deal of attention has been focused on porous carbon materials with high surface area in different nanostructures, especially the mesopores or hierarchical porous confinements in nanoscale [16,17,18]. This is due to their strong interactions with special sites or dangling bonds in carbon, resulting in higher total capacity than theoretical value for LiC_6_ intercalacted stoichiometry in graphitic structures [19,20,21]. Moreover, optimal micro/mesopores with high surface area interconnected with macroporous is an efficient mass transfer pathway for Li ion diffusion. Additionally, such structures composed of interlaced networks of interconnecting pores and carbon framework do not require expensive production costs for commercialization [13,22,23].

In this regard, the ordered mesoporous carbon spheres are in particular interesting because of their nano-sized spherical architecture, surface functionality and tunable particle size to facilitate rapid charge transfer and minimize polarization effects [14,24,25,26]. A template carbonization method is one of the most powerful and useful for tunable size in precise range, where nanostructured and/or self-assembled materials are supported as the templates [27,28,29,30,31]. Ordered mesoporous carbon nanospheres (OMCS) can be successfully synthesized by the carbonization of suitable carbon precursors by organic-organic assembly method with novel low-concentration hydrothermal route demonstrated by Zhou *et al.* [24]. It is reported that a highly ordered mesoporous carbon nanoparticles with spherical morphology and uniform size can be applied for low cytotoxic and excellent cell permeability applications. However, such useful carbon spheres have not been evaluated for application as anode in lithium ion batteries. In this paper, we examined a development of OMCS of different sizes by low-concentration hydrothermal approach and their application as an anode material in LIBs.

## 2. Results and Discussion

**Figure 1:**
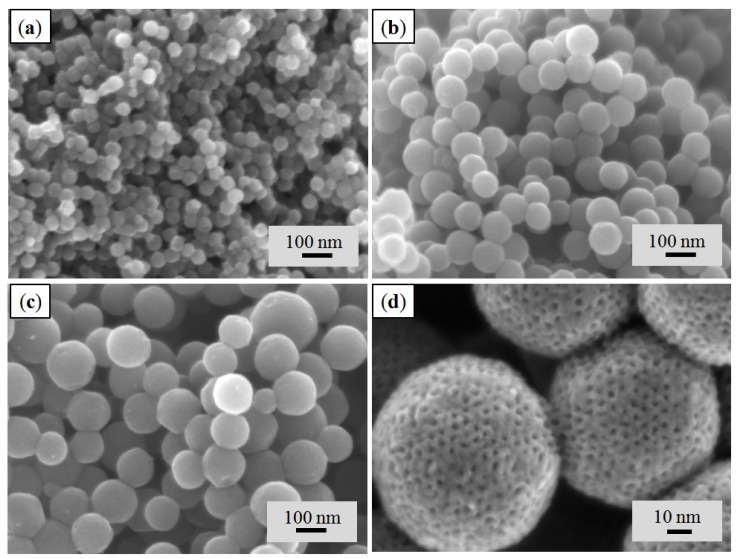
Scanning electron microscopy (SEM) images of (**a**) OMCS_7_, (**b**) OMCS_9_, (**c**) and (**d**) OMCS_11_ obtained by dilution of phenolic resol/F127 to different volumes followed by hydrothermal method and calcination at 700 °C in N_2_ atmosphere.

The ordered mesoporous carbon nanospheres have been synthesized successfully in various diameters by typical hydrothermal method and abbreviated as OMCS*_x_* (*x* = volume of prepared phenolic resol/F127 solution in mL). SEM images displayed in Figure 1 confirmed the formation of uniformly spherical structures of OMCS_7_, OMCS_9_ and OMCS_11_ with average diameters of 46 ± 3, 95 ± 5 and 130 ± 19 nm, respectively (Figure 1a–c). The high resolution SEM image of OMCS_11_ (Figure 1d) indicated the presence of ordered mesopores and open pore structure over the carbon nanospheres. In addition, the average pore size was noted to be around 2.5 nm. In addition, the OMCS_11_ has a relatively wide particle size distribution (~15% standard deviation) compared to others synthesized in the present work.

**Figure 2:**
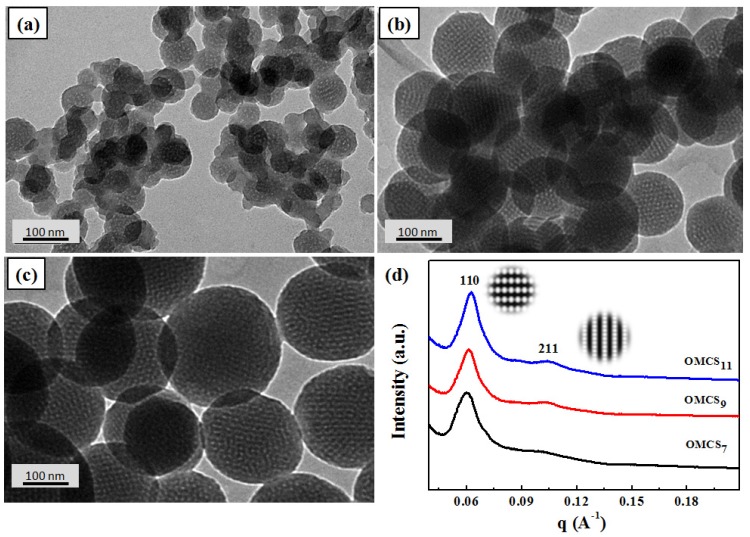
Transmission electron microscopy (TEM) images of (**a**) OMCS_7_, (**b**) OMCS_9_, (**c**) OMCS_11_, and (**d**) small-angle scattering system (SAXS) patterns of OMCS*_x_*.

Further morphological characterization has been performed by recording TEM and SXAS presented in Figure 2. Two directions, {111} and {110} of OMCS*_x_* illustrates the formation of body-centered cubic pore-structures. Additionally, pore size of OMCS*_x_* is noted to be 2.6 nm, which is similar to the estimated value from ultra-high resolution SEM images and is in good agreement with the findings in the literature [24]. It is noteworthy that the SAXS patterns of OMCS*_x_* exhibit two obvious {111} and {211} reflection peaks with *q* values of 0.061 and 0.11 Å^−1^, which correspond to the d-spacings of 10 and 5.7 nm, respectively. The *q* ratio of 1:3^0.5^ clearly indicates the periodic mesoporous structure with [Im3m] symmetry of OMCS*_x_*.

**Figure 3:**
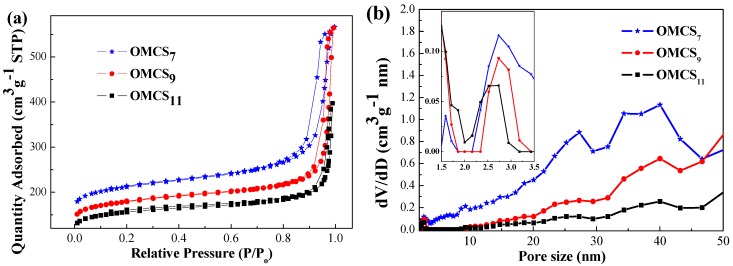
(**a**) The N_2_ adsorption-desorption isothermals and (**b**) pore size distributions of OMCS*_x_*.

BET analysis of OMCS_7_, OMCS_9_ and OMCS_11_ were performed and the results are displayed in Figure 3. The surface areas of OMCS were calculated from N_2_ sorption isotherms, and the specific surface areas of OMCS_7_, OMCS_9_ and OMCS_11_ were 728, 615, and 537 m^2^·g^−1^, respectively. A previous study used template method to fabricate highly ordered mesoporous carbonaceous materials and found that the specific surface areas of the body-centered cubic OMC with space group of [Im3m] were in the range of 580–600 m^2^·g^−^^1^ [32], which is comparable with the result obtained in this study. However, the specific surface areas obtained in this study are lower than some reported data [24], presumably due to the linkage of inter-particles and the decrease in active sites for N_2_ adsorption. Additionally, the average pore size and pore volume are in the range of 2.7–5.0 nm and 0.45 to 0.82 cm^3^·g^−1^, respectively. The typically small mesopores in an indistinct capillary condensation step in the *P*/*P*_0_ region of 0.2–0.4 and interparticle texture between the carbon spheres at *P*/*P*_0_ 0.9–0.995 resulted in each pseudo-type-I curve with H1 hysteresis loop of OMCS*_x_* [24]. Remarkably, OMCS_7_ displays the thickest hysteresis loop and then decreases with the increase in diameters of OMCS*_x_* (OMCS_9_ and OMCS_11_).

Table 1 shows the specific surface areas and pore textures of OMCS*_x_*. It is clear that the average surface area (*S*_BET_) and *t*-plot micropore surface area of OMCS are decreased upon increasing particle size of OMCS. The negative correlation between the particle size of carbon nanosphere and specific surface area is mainly due to the fact that small particle size can increase in inter-spherical textures and the pore volumes. Although the increase in pore size favors facile Li diffusion through the mesopore to access more surface active sites for Li ion adsorption [33], the cavity effect, which has been developed for almost a decade, can be used to explain the high specific capacity for lithiation in the pore size range of 0.5–1.5 nm [34]. It is noteworthy that the ratios of micropore surface area to BET surface area of OMCS*_x_* are similar (0.691–0.697) and the OMCS_11_ exhibits a high peak at around 1.5 nm, small pore size of 2.7 nm and wide pore size distribution in the range of 1.5–2.5 nm (Figure 3b), suggesting that the electrochemical performance of OMCS_11_ may be superior when compare with those of OMCS_7_ and OMCS_9_.

**Table 1:** The pore texture and specific surface area of OMCS*_x_*.

Sample	*S*_BET_^a^ (m^2^·g^−1^)	Pore Size ^b^ (nm)	Pore Volume ^b^ (cm^3^·g^−1^)	*t*-Plot Micropore Surface Area (m^2^·g^−1^)	Micropore/Total Surface Area Ratio
OMCS_7_	728	5.0	0.82	505	0.694
OMCS_9_	615	3.4	0.67	425	0.691
OMCS_11_	537	2.7	0.45	374	0.697

^a^ The Brunauer-Emmett-Teller method was utilized to calculate the specific surface area using adsorption data in the relative pressure (*P*/*P*_0_) range of 0.04–0.2; ^b^ By using the density functional theory (DFT) model, the pore volumes and pore size were calculated from the adsorption branches of isotherms, and the pore volume was estimated from the adsorbed amount at a relative pressure (*P*/*P*_0_) of 0.996.

**Figure 4:**
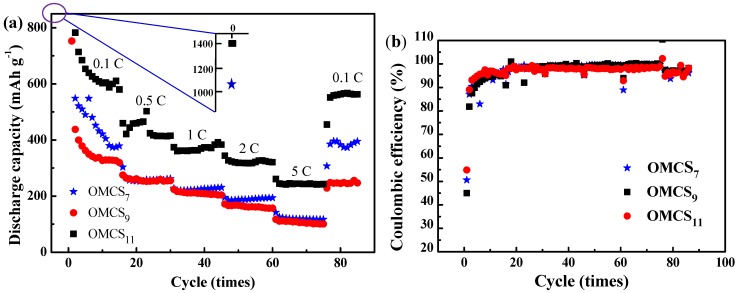
The Li-ion batteries (LIB) half cell performances of (**a**) discharge capacity and (**b**) coulombic efficiency of OMCS*_x_* under various *C* rates from 0.1 *C* to 5 *C*.

The electrochemical performance of as-synthesized OMCS*_x_* were evaluated as an anode for LIB half-cell in the voltage range of 0.05–3 V at different *C* rates from 0.1 *C* to 5 *C*. As shown in Figure 4, the initial discharge capacity of OMCS_7_, OMCS_9_ and OMCS_11_ at 0.1 *C* were noted to be 1018, 752 and 1421 mA·h·g^−1^, respectively. The experimental specific capacities are higher than that of typical graphite, presumably due to the presence of mesoporous structures [25,35,36]. Furthermore, the capacity of OMCS*_x_* decreased gradually in the first 10 cycles, and then stabilized well during the subsequent cycles. The decrease in capacity at the first several cycles is attributed to the decomposition of electrolytes and formation of solid-electrolyte interface (SEI) between inter-spherical textures, especially for the small size of OMCS_7_. After 15 cycles at the same *C*-rate, OMCS_7_, OMCS_9_ and OMCS_11_ delivered stable capacities of 378, 319 and 580 mA·h·g^−1^ with high coulombic efficiency of >95%.

It is interesting to note that OMCS_11_ with small pore size and relatively wide particle size distribution delivers a superior performance compared with those of OMCS_7_ and OMCS_9_, indicating the combination effect of pore size and particle size distribution on the high electrochemical performance of OMCS_11_. The electrochemical results show that using OMCS_11_ as the anode material exhibits enhanced rate performance (by ≈150%) compared to OMCS_7_ and OMCS_9_ at various *C*-rates. The discharge capacities at specific *C*-rate (Figure 4) were 415, 383, 320 and 243 mA·h·g^−1^ at 0.5 *C*, 1 *C*, 2 *C* and 5 *C*, respectively. Such commendable performance may be attributed to the ordered mesoporous spherical structure with wide particles distribution. This results in the development of intimate contact with carbon black because of the existence of various sizes of porous carbon nanospheres. Therefore, the large particle size of OMCS_11_ can maintain sufficient space for rapid transport of electrolyte and the relatively wide particle size distribution provides closer contact between OMCS and conductive materials, leading to the effective electron transfer from current collector to active material (OMCS). Furthermore, the fast diffusion rate for Li ions increases the capacity and rate performances significantly [14,37,38]. In addition to OMCS*_x_*, several studies have depicted that that the introduction of graphene nanomaterials exhibited high reversible capacity (540–1264 mA·h·g^−^^1^) and good cyclic performance on LIBs because of the high surface area and large mesopore volume [39,40], suggesting that the 3D graphene nanomaterials may be promising anode materials for LIB application.

**Figure 5:**
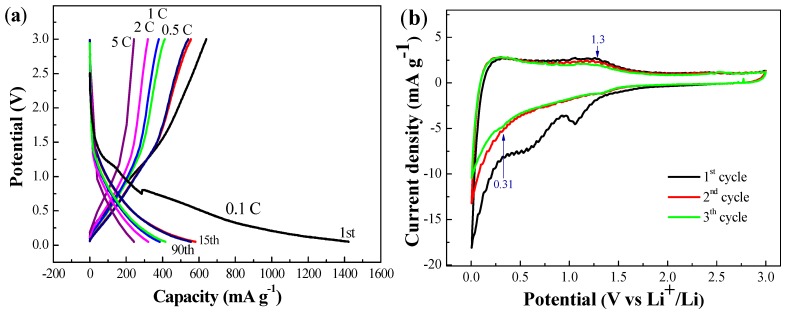
(**a**) Galvanostatic discharge-charge profiles under various *C*-rate from 0.1 *C* to 5 *C*; and (**b**) cyclic voltammograms in the potential window of 0.01–3 V (*vs.* Li^+^/Li) at a scan rate of 0.1 mV·s^−1^ of OMCS_11_.

The galvanostatic discharge-charge profiles (Figure 5a) of OMCS_11_ have been recorded at 0.1–5 *C* in the potential window of 0.05–3.0 V (*vs.* Li^+^/Li). The specific capacity at the end of cycles for each current rate is also shown. The plateau decreases slightly after 0.5 V, which can be attributed to the formation of SEI layer, leading to the irreversible capacity. At higher current rates, no obvious plateaus could be identified due to typical properties of porous carbon electrodes [18,25]. The cyclic voltammogram (CV) of OMCS_11_, as shown in Figure 5b, was recorded at a scan rate of 0.1 mV·s^−1^ in the potential window of 0.01–3 V (*vs.* Li^+^/Li) for the first three cycles. In addition, two peaks at 0.58 and 1.06 V appeared in the first cycle of cathodic process and then diminished in the subsequent cycles. This indicates the irreversible formation of SEI layer in the inter-particles of OMCS_11_. In addition, a broad peak at 0.31 V was noted, which extended to 0.01 V in the following cycles. This in all probability may correspond to the reversible lithiation of amorphous graphitic carbon structures. Alternatively, the peak at 1.3 V in anodic polarization process represents the delithiation reaction of LiC_6_ structures.

**Figure 6:**
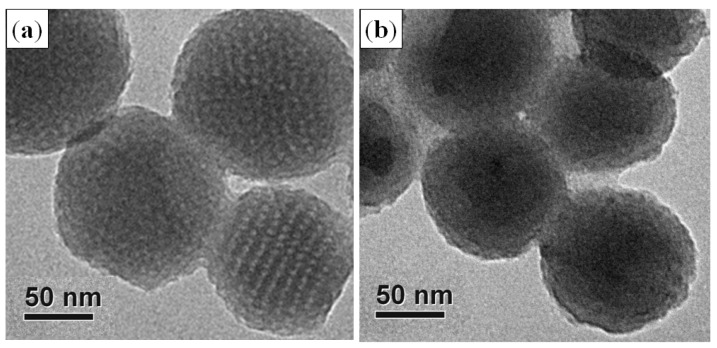
TEM images of OMCS_11_ electrodes (**a**) before and (**b**) after assigned charging-discharging processes under various *C*-rate from 0.1 *C* to 5 *C* for 85 cycles.

TEM images were recorded after cycling at specific *C*-rate for 85 cycles (Figure 6), which show the spherical structures of OMCS_11_ was maintained with the formation of SEI coating. The mesoporous structures are faintly observable at the fringes of carbon spheres rather than at the central regions due to the blurring of SEI layer.

## 3. Experimental Section

### 3.1. Chemicals

All the chemicals used in this work were of analytical grade and used without further purification. Phenol (99%) and methanal (24% aqueous solution) were purchased from Acros (Geel, Belgium). Amphiphilic triblock copolymer Pluronic F127 (*M_W_* = 12600, PEO_106_PPO_70_PEO_106_), polyvinylidene fluoride (PVDF) and *N*-methylpyrrolidone (NMP) was obtained from Sigma-Aldrich (St. Louis, MO, USA). Carbon black (primary particle 13 nm) was the product of Uni-Onward Co. (Hsinchu, Taiwan). The double distilled deionized water (Millipore, 18.3 MΩ·cm) was used as solvent throughout the experiments.

### 3.2. Synthesis of Ordered Mesoporous Carbon Nanospheres (OMCS)

OMCS were synthesized following basic polymerization assisted hydrothermal synthesis [1], using the low-molecular-weight polymer (resol) (*M_W_* = 500–5000) as the carbon source and pluronic F127 as the surfactant. In a typical procedure, 0.6 g of phenol, 3.4 mL of methanal (24%) and 15 mL of 0.1 N NaOH_(aq)_ solution were mixed and vigorously stirred at 70 °C. After 30 min, the aqueous solution of F127 (0.96 g of F127 dissolved in 15 mL of water) was added gradually to the above mixture. Subsequently, after 3 h of stirring, 50 mL of distilled water was added. This resulted in the formation of phenolic resol/F127 mono-micelles after 12–15 h with the appearance of muddy solution in red color. On standing, the appropriate amount of transparent liquor were collected and assigned as *x* = 7, 9, 11 mL of phenolic resol/F127 solution and diluted to 35 mL as the final volume. The diluted phenolic resol/F127 solution was transferred into an autoclave and heated at 130 °C for 24 h. The products obtained from the hydrothermal reaction were washed by DI water for three times, centrifuged and dried at 40 °C for 12 h. Finally, the dried samples were heated from 60 to 700 °C at a rate of 1 °C·min^−1^ and maintained at 700 °C for 2 h in N_2_.

### 3.3. Characterization and Measurements

The electron microscopy (EM) images were collected using: (i) JEOL JSM-6700F SEM (Tokyo, Japan); (ii) Hitachi SU8220 ultra-high resolution SEM (Tokyo, Japan) and (iii) JEOL-2010 TEM (200 kV, Tokyo, Japan). N_2_ adsorption-desorption isotherms were recorded at 77 K using Micromeritics 2020 analyzer (Norcross, GA, USA). The small angle X-Ray scattering (SAXS) measurements were conducted using X-ray scattering instrument (Hsinchu, Taiwan) with a superconducting wiggler insertion device at the BL23A beamline in the National Synchrotron Radiation Research Center, Hsinchu, Taiwan. Wide-angle XRD patterns were recorded on a Bruker D8 X-ray diffractometer (Karlsruhe, Germany) with Ni-filtered Cu Kα radiation (λ = 1.5406 Å).

### 3.4. Electrochemical Measurements

The electrochemical measurement of OMCS*_x_* electrodes were analyzed using 80 wt % of respective OMCS*_x_* samples, mixed with 10 wt % carbon black particles as conductive additive, and PVDF as binder in NMP solvent into homogeneous slurry. The slurry was then spread onto a copper foil current collector at a thickness of 50 μm by a glass rod with 1.5 mg of active material. The slurry was then pressed into a spherical electrode with the diameter of 1 cm, and then dried in vacuum at 100 °C. Electrochemical test cells were assembled by using the CR-2032 coin cell in a N_2_-filled glovebox and the coated copper foil was applied as the working electrode and Li metal foil as counter electrode. The electrolyte was composed of 1.15 M solution of LiPF_6_ in a 3:4:2 (*v*/*v*/*v*) mixture of ethylene carbonate (EC), ethyl methyl carbonate (EMC) and dimethyl carbonate (DMC). The cells were charged and discharged galvanostatically in terms of 0.1, 0.5, 1, 2 and 5 *C* (1 *C* = 372 mA·h·g^−1^) in fixed voltage window of 0.05–3 V and cyclic voltammograms were recorded in the potential window of 0.01–3 V (*vs.* Li^+^/Li) at a scan rate of 0.1 mV·s^−1^ by a Maccor battery testing system (Tusla, OK, USA) at room temperature.

## 4. Conclusions

In the present work, three different sizes of ordered mesoporous carbon nanospheres were successfully synthesized by hydrothermal treatment followed by calcination of the as-prepared phenolic resol/F127 solution. The diameters of OMCS_7_, OMCS_9_ and OMCS_11_ are 46 ± 3, 95 ± 5 and 130 ± 19 nm, respectively. It is noted that the positive correlation between the average sizes of OMCS*_x_* and its standard deviation are crucial factors for enhanced performance as anode materials in LIBs. OMCS_11_ delivers an excellent capacity retention of 560 mA·h·g^−1^ at 0.1 *C* after 85 cycles as well as an outstanding rate capability of 240 mA·h·g^−1^ at 5 *C* for 15 cycles. This performance is significantly superior compared with other ordered mesoporous carbon nanospheres prepared in the present work. In addition, the structural and morphological integrity of the as-prepared anode material can be maintained even after continuous cycling at various current rates. This is mainly attributed to the suitable size distribution and formation of optimal contact between OMCS_11_ and conductive additive which facilitates the effective charge transfer.
